# Pharmacological insights into *Arthrospira platensi*s (Oscillatoriaceae): Ethnopharmacology, mechanisms, and therapeutic potential in smooth muscle disorders

**DOI:** 10.3389/fphar.2025.1653808

**Published:** 2025-10-30

**Authors:** Anderson Fellyp Avelino Diniz, Bárbara Cavalcanti Barros, João Marcos Araújo da Silva, Ray Ravilly Alves Arruda, Brena Freire de Oliveira Claudino, Michel Benício de Melo, José Edvaldo Cavalcanti de Sousa Filho, Francisco Fernandes Lacerda Júnior, Maxsyara Felismino da Silva Soares, Thais Rosa de Sousa, Paula Benvindo Ferreira, Bagnólia Araújo da Silva

**Affiliations:** ^1^ Postdoctoral Researcher in the Postgraduate Program in Natural and Synthetic Bioactive Products Health Sciences Center, Federal University of Paraiba, João Pessoa, Brazil; ^2^ Postgraduate Program in Natural and Synthetic Products Bioactive/Health Sciences Center, Federal University of Paraiba, João Pessoa, Brazil; ^3^ Health Sciences Center, Federal University of Paraiba, João Pessoa, Brazil; ^4^ Applied Cellular and Molecular Biology Program, University of Pernambuco, Recife, Brazil; ^5^ Coordinator of the Clinical Pharmacy at the Hospital do Servidor General Édson Ramalho, João Pessoa, Brazil; ^6^ Pharmaceutical Sciences Department/Health Sciences Center/Federal University of Paraiba, João Pessoa, Brazil

**Keywords:** *Arthrospira platensis*, smooth muscle, oxidative stress, inflammation, therapeutic nutrition, functional food

## Abstract

Arthrospira platensis (Oscillatoriaceae) (AP): commonly known as Spirulina, is a widely cultivated cyanobacterium used as both a dietary supplement and a functional food. Growing evidence suggests potential therapeutic effects in smooth muscle–related disorders; however, critical evaluations of the available data remain scarce. This narrative review critically examines preclinical and clinical evidence on *Arthrospira platensis* and its bioactive metabolites in conditions involving smooth muscle dysfunction, highlighting methodological strengths and limitations, and outlining future research needs. A structured literature search was conducted in PubMed, Scopus, and Web of Science using predefined inclusion criteria, and only studies with validated taxonomy and experimental or clinical data were included. The GA-online Best Practice checklist and the Four Pillars of Best Practice in Ethnopharmacology guided the analysis. Preclinical studies consistently demonstrate antioxidant, anti-inflammatory, and smooth muscle–modulating effects of *A. platensis* extracts and metabolites, including phycocyanin and polysaccharides. Experimental models in vascular, intestinal, uterine, and airway tissues reveal improved contractility and reduced oxidative damage. Although limited, clinical evidence suggests benefits on metabolic parameters and cardiovascular risk factors. Major limitations include the absence of standardized extract characterization, variable dosing, inconsistent controls, and the scarcity of randomized clinical trials. In conclusion, *Arthrospira platensis* shows promising pharmacological activities relevant to smooth muscle physiology, but current evidence remains largely preclinical and constrained by methodological weaknesses. Standardized extract characterization, rigorous experimental designs, and adequately powered clinical trials are essential to confirm its therapeutic potential.

## 1 Introduction

Smooth muscle tissue is widely distributed throughout the human body and represents the primary muscle type regulating the function of most hollow organs, including the uterus, blood vessels, bladder, airways, stomach, and intestines (Webb, 2003). In the reproductive system, it is essential for fertility, sexual function, and urination. Within the vascular system, smooth muscle plays a critical role in tissue oxygenation and blood pressure regulation. In the urinary system, it contributes to electrolyte balance and toxin elimination, while in the gastrointestinal tract it is fundamental for digestion, nutrient absorption, and interaction with the microbiota (Palmisano et al., 2017; Hafen and Burns, 2018).

Unlike skeletal muscle, smooth muscle functions involuntarily and is regulated by the autonomic nervous system via hormones, neurotransmitters, and receptor-mediated signaling. Dysfunction of smooth muscle contributes to the pathogenesis of various disorders, including asthma, gastrointestinal diseases, reproductive dysfunction, and hypertension. Calcium ions (Ca^2+^) are central mediators of both physiological and pathological smooth muscle contraction, as well as targets of the pharmacological action of numerous agents (Williams D. M. and Rubin B. K., 2018; Kim et al., 2008).

*Arthrospira platensis* (Oscillatoriaceae) (*AP*), commonly known as Spirulina, is a photosynthetic cyanobacterium that can occur in unicellular or multicellular filamentous forms (Conradie et al., 2008; Papini et al., 2021; Chaiklahan et al., 2013; Maddiboyina et al., 2023). Recognized as a “superfood,” AP is rich in diverse bioactive metabolites, including vitamins, pigments, minerals, proteins, carbohydrates, essential fatty acids, amino acids, phenolics, glycosides, flavonoids, and alkaloids (Hayashi et al., 1996; Maddina et al., 2016; Hasanein et al., 2018; Prabakaran et al., 2020). Its lipid profile is characterized by a high proportion of polyunsaturated fatty acids (PUFAs), particularly linolenic acid, which accounts for approximately 36% of its total fatty acid content (Demir and Tükel, 2010; Diniz et al., 2024).

Due to its high nutritional value, AP is among the most widely consumed microalgae worldwide (Li et al., 2022; Omar et al., 2022). Its characteristic spiral-shaped filaments, composed of complex sugars and proteins, make it particularly suitable as a dietary supplement and functional food (Bortolini et al., 2022; Gentscheva et al., 2023; Dhandwal et al., 2024; Terzioğlu et al., 2024).

**FIGURE 1:**
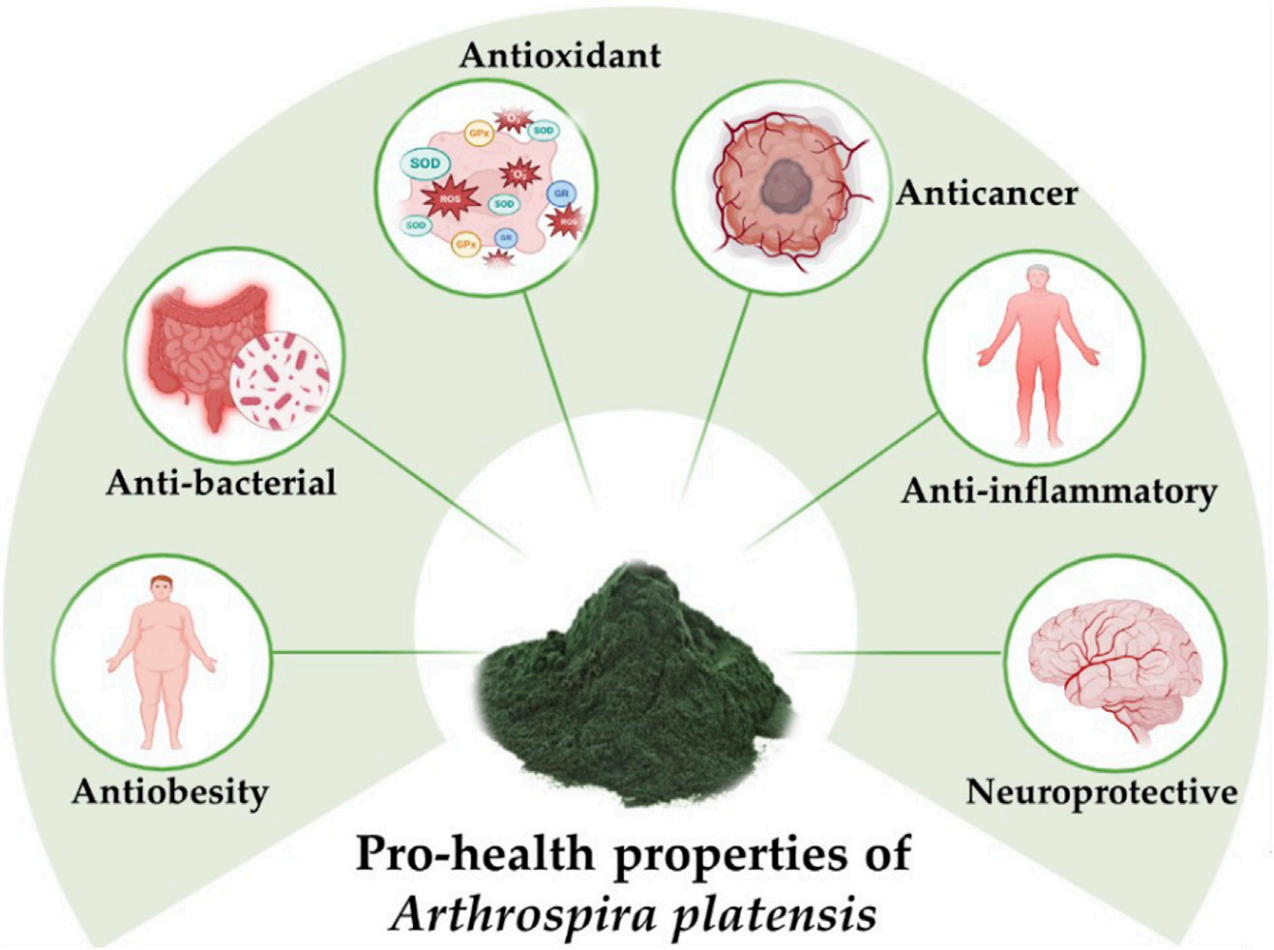
Prohealth properties of *Arthrospira platensis*. The bioactive metabolites of this cyanobacterium exhibit a wide range of pharmacological effects, including antioxidant, anticancer, anti-inflammatory, neuroprotective, anti-obesity, and antibacterial activities.

The biological and pharmacological activities of AP have been extensively documented, supporting its health-promoting potential (Chwil et al., 2024; Pradhan et al., 2022; Jungclaus et al., 2023; Hidhayati et al., 2022). Reported effects include anti-viral, antibacterial, antifungal, anti-inflammatory, antioxidant, photoprotective, neuro-protective, anti-aging, anticancer, and anti-obesity activities (Abbas et al., 2021; Hamad et al., 2023; Calella et al., 2022; Perna et al., 2023; Kumar et al., 2014; Kaipa et al., 2022; Ansari et al., 2023; Stunda-Zujeva et al., 2023). Its therapeutic applications have been investigated in cardiovascular diseases (Wang et al., 2020; Prete et al., 2024), gastrointestinal disorders and dysbiosis (Asmaz and Seyidoglu, 2022; Liu et al., 2021; Diniz et al., 2023), hypertension (Ghaemfar et al., 2021; Otero and Verdasco-Martín, 2023), diabetes (Mazloomi et al., 2022; Ahda et al., 2024), Alzheimer’s disease (Tamtaji et al., 2023; Tavares et al., 2024), infectious diseases (Brito et al., 2020; Abu-Taweel et al., 2019; Ratha et al., 2021), cancer (Ge et al., 2019; Hamdy et al., 2024), and sexual dysfunction (Diniz et al., 2020; Souza et al., 2022) (Figure 1).

In this context, *A. platensis* has attracted attention for its applications in the development of bioactive metabolites, pharmaceuticals, cosmetics, fuels, and nutritional products. Nevertheless, few studies have specifically addressed its therapeutic potential in the prevention or treatment of disorders associated with smooth muscle dysfunction. This review seeks to fill this gap by providing an updated and comprehensive synthesis of preclinical and clinical evidence on the bioactive effects of *A. platensis*, with emphasis on conditions involving smooth muscle across multiple physiological systems. “This review was structured according to the Four Pillars of Best Practice in Ethnopharmacology (Frontiers in Pharmacology), ensuring taxonomic validation of *A. platensis* Gomont (Oscillatoriaceae), characterization of its bioactive metabolites, assessment of traditional and current uses, and a critical appraisal of pharmacological and clinical studies.”

## 2 Methods

This narrative review was conducted in accordance with the GA-online Best Practice tool (https://ga-online.org/best-practice/) and the Four Pillars of Best Practice in Ethnopharmacology (https://www.frontiersin.org/files/pdf/4_pillars_FULL_TEXT.pdf).

### 2.1 Literature search strategy

A structured literature search was performed to identify studies addressing the therapeutic potential of *Arthrospira platensis* (Gomont) Kützing ex Gomont, Cyanobacteriaceae in smooth muscle–related disorders. The databases PubMed, Scopus, Web of Science, and SciELO were searched for articles published between 2010 and 2025. Reference lists of retrieved articles and relevant reviews were also screened for additional studies. The literature search was performed in PubMed, Scopus, and Web of Science using the following keywords: “Arthrospira platensis,” “Spirulina platensis,” “smooth muscle,” “oxidative stress,” “antioxidant,” “inflammation,” “uterine contraction,” “vascular,” “intestinal,” “airway,” “pharmacology,” and “clinical trial.” Boolean operators (AND/OR) were applied to refine the search strategy.

### 2.2 Eligibility criteria and study selection

Inclusion criteria were: (i) validated taxonomy of *A. platensis*; (ii) experimental data from *in vitro* or *in vivo* pharmacological models, or clinical trials; and (iii) publication in English. Exclusion criteria were: review articles, conference abstracts, studies lacking pharmacological data, or those using non-validated taxa.

**FIGURE 2:**
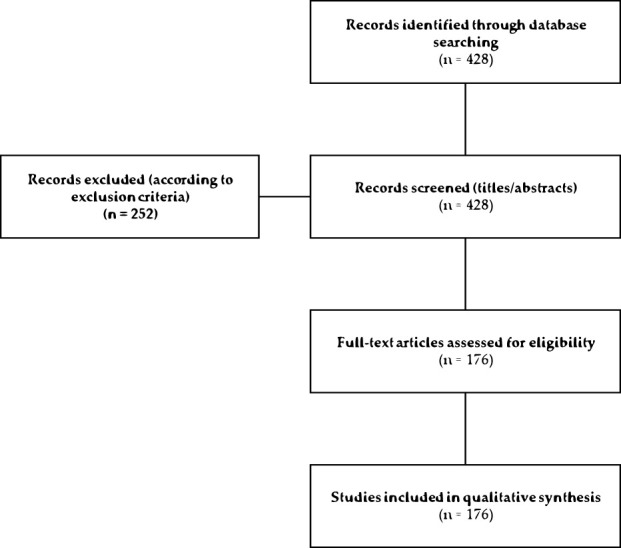
PRISMA flowchart adapted from Page *et al.* (2021), summarizing the literature search and study selection process. A total of 428 records were identified through database searching. After title and abstract screening, 252 records were excluded. A total of 176 full-text articles were assessed for eligibility and were included in the qualitative synthesis (Page et al., 2021).

The search initially retrieved 428 articles. After screening titles and abstracts, 252 articles were excluded, and 176 studies were included in the qualitative synthesis. The selection process is summarized in the PRISMA flowchart (Figure 2).

## 3 Physiology and functional role of smooth muscle

Mammals possess three primary types of muscle: skeletal, cardiac, and smooth. Skeletal. muscle is attached to bones and provides structure and strength; cardiac muscle is found in the heart and enables blood circulation; and smooth muscle controls the function of most hollow organs and tubular structures throughout the body. Smooth muscle is located in all blood vessels, the gastrointestinal (GI) tract, bronchioles, uterus, and bladder. Unlike skeletal and cardiac muscle, smooth muscle lacks striations and sarcomeres and operates involuntarily through reflexes and autonomic nervous system (ANS) control. Smooth muscle fibers differentiate from splanchnic mesoderm (Webb, 2003; Kuo and Ehrlich, 2015).

Microscopically, smooth muscle appears homogeneous; however, it is rich in actin and myosin filaments, which play essential roles in excitation-contraction coupling. Smooth muscle contraction is primarily regulated by hormones, autocrine/paracrine agents, and chemical signals. There are two main types of smooth muscle: unitary and multi-unit. Unitary smooth muscle cells are interconnected by gap junctions (connexins), allowing synchronous contraction from a single synaptic input—these are found in the intestines and blood vessels. In contrast, multi-unit smooth muscle cells receive individual synaptic inputs and exhibit more controlled and graded responses, typical in the eye and hair follicles (Kuo and Ehrlich, 2015; Noto and Edens, 2019; Gash et al., 2023).

Each muscle type has unique cellular components, pathophysiological characteristics, and specific functions. Skeletal muscle accounts for approximately 40% of total body weight and comprises multiple fibers bundled into muscle spindles, which act as functional units enabling contraction and relaxation (Frontera and Ochala, 2015). Skeletal muscle contraction relies on membrane depolarization, which links excitation to calcium release from the sarcoplasmic reticulum (SR) (Schneider and Chandler, 1973; Nelson et al., 1995; Savalli et al., 2021; Wu et al., 2021).

Cardiac muscle is composed of striated fibers under involuntary control via the ANS. It contains individual cardiomyocytes and specialized pacemaker cells located within the myocardium. Cardiac contraction is regulated by Ca^2+^-induced Ca^2+^ release from the SR, triggered by extracellular Ca^2+^ influx (Barcenas-Ruiz and Wier, 1987; Cann et al., 1987; Moss et al., 2021; Burkhard et al., 2017; Mijailovich et al., 2021; Greenberg et al., 2021).

In contrast, smooth muscle contraction is not under voluntary control and is regulated autonomously through calcium–calmodulin interaction. Contraction is initiated by changes in membrane potential or by activation of mechanosensitive receptors located in the plasma membrane (Webb, 2003). Accordingly, layers of smooth muscle cells play a critical role in both physiological and pathological processes, primarily due to the significant and interdependent effects of various signaling pathways that modulate smooth muscle activity (Giuseppe and Paul, 2015; Williams D. A. and Rubin L. J., 2018). Given the widespread distribution of smooth muscle throughout the body, abnormalities in its contraction or regulation are associated with a broad spectrum of pathological conditions.

**FIGURE 3:**
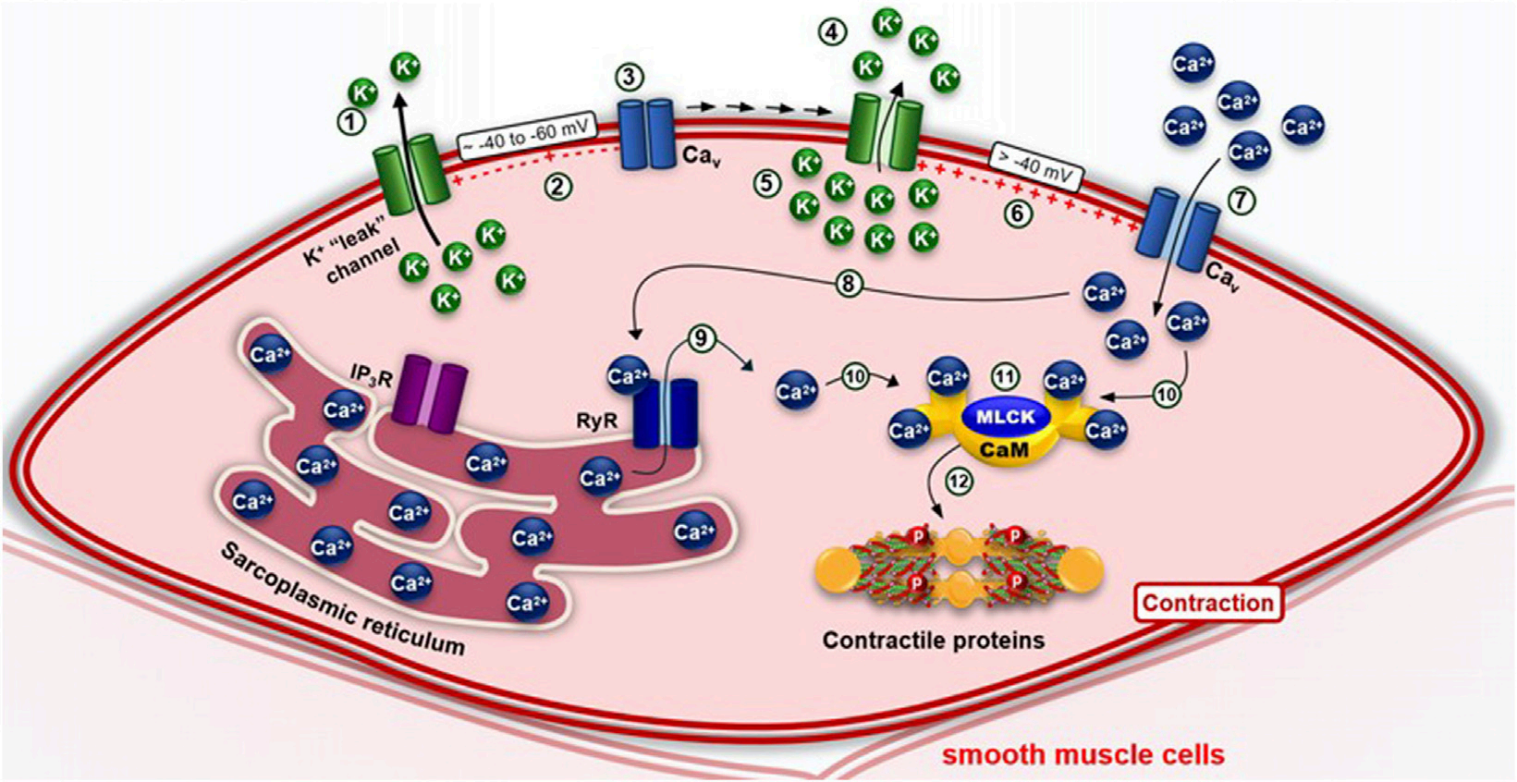
(1) At rest, the electrochemical gradient favors the efflux of K^+^ ions through their leak channels; (2) this maintains the inner perimembranous region of smooth muscle cells in a negatively polarized state, (3) thereby keeping the voltage-dependent L-type calcium channels (Ca_V_ L) closed. (4) An increase in extracellular [K^+^] reduces K^+^ efflux, (5) resulting in K^+^ accumulation in the cytosol, (6) which depolarizes the perimembranous region. (7) This change in membrane potential opens Cav L channels, allowing Ca^2+^ influx. (8) The increase in intracellular Ca^2+^ activates ryanodine receptors (RyR), (9) triggering the release of Ca^2+^ from the sarcoplasmic reticulum. (10–11) The elevated [Ca^2+^]c leads the formation of the [4Ca^2+^–CaM] complex, which activates myosin light chain kinase (MLCK). (12) MLCK-mediated phosphorylation of contractile filaments results in smooth muscle cell contraction.

**FIGURE 4:**
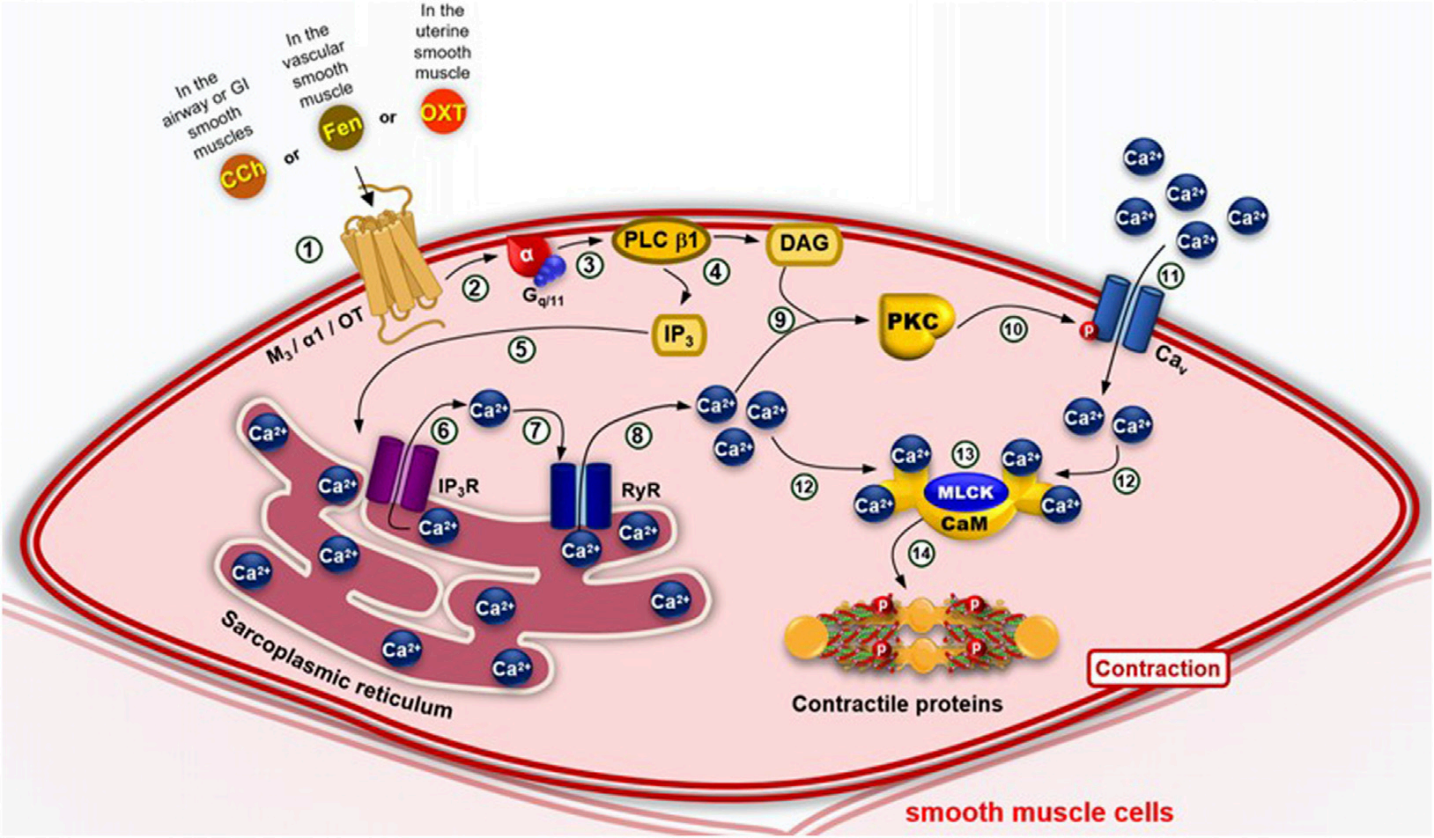
Pharmacomechanical coupling of smooth muscle contraction via the G_q/11_–PLC β1 pathway. (1) Agonists–such as carbachol binding to the M3 receptor (on airway and GI smooth muscle), phenylephrine binding to the α1 receptor (on blood vessel smooth muscle) or oxytocin binding to the OT receptor (on uterine smooth muscle) - inducing a conformational change that recruits the G_q_ or G_11_ proteins, exchanging GDP for GTP and causing steric hindrance that dissociates the α-GTP subunit from the βγ dimer. (2–3) The α-GTP subunit activates phospholipase C β1 (PLC β1). (4) This enzyme hydrolyzes phosphatidylinositol 4,5-bisphosphate (PIP_2_) into inositol 1,4,5-trisphosphate (IP_3_) and diacylglycerol (DAG). (5) Soluble IP_3_ diffuses through the cytosol and activates the IP_3_ receptor (IP_3_R), (6) promoting Ca^2+^ release from the sarcoplasmic reticulum. (7) Ca^2+^ also binds to the ryanodine receptor (RyR), (8) leading to further calcium release into the cytosol. (9) Ca^2+^ activates calcium-dependent protein kinase (PKC), exposing the DAG-binding site. (10) PKC translocates to the membrane, binds DAG, and becomes active. (11) PKC phosphorylates voltage-dependent calcium channels (Ca_V_), (12) facilitating additional Ca^2+^ influx. (13) Elevated [Ca^2+^]c promotes formation of the Ca^2+^–calmodulin (CaM) complex. (14) Which activates myosin light chain kinase (MLCK). This leads to phosphorylation of contractile filaments and smooth muscle contraction.

In this context, smooth muscle contraction is independent of the troponin complex, relying instead on calcium influx. This distinction lies in how calcium enters the cell and raises cytosolic calcium concentration, a process that can occur through three main mechanisms (Somlyo and Somlyo, 2003; Dirksen et al., 2022):

1. *Membrane depolarization (electromechanical coupling):* Depolarization of the cell membrane results in an increase in cytosolic calcium concentration ([Ca^2+^]c). This occurs through the activation of voltage-dependent calcium channels (Ca_V_), which open in response to the depolarization and allow calcium influx into the cell (Figure 3).
2. *Agonist-induced activation of G protein-coupled receptors (pharmacomechanical coupling):* Agonists such as drugs, hormones and/or neurotransmitters—for example, carbachol (in airway and gastrointestinal smooth muscle), phenylephrine (in vascular smooth muscle), and oxytocin (in uterine smooth muscle)—can trigger the phospholipase C β1 (PLC β1) pathway by activation of their receptors, caus-ing calcium release from the sarcoplasmic reticulum mediated by inositol 1,4,5-trisphosphate (IP_3_) and, subsequently, an influx of these ions through Cav in the cell membrane, in processes called phasic and tonic contraction, respectivel (Figure 4).

**FIGURE 5:**
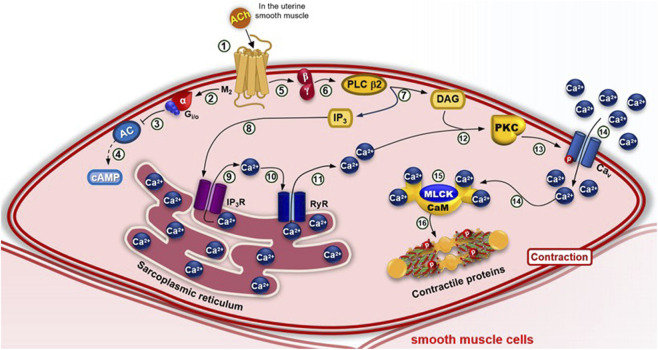
Pharmacomechanical coupling of smooth muscle contraction via the G_i/o_–PLC β2 pathway. (1) In some types of smooth muscle, such as uterine, agonists such as acetylcholine bind to M2 receptors, triggering a conformational change that recruits G_i_ or G_o_ proteins, exchanging GDP for GTP and dissociating the α-GTP subunit from the βγ dimer. (2–3) The α-GTP subunit inhibits adenylyl cyclase (AC), (4) reducing cytosolic cAMP levels. (5–6) The βγ dimer activates phospholipase C β2 (PLC β2), (7) which hydrolyzes PIP_2_ into IP_3_ and DAG. (8–9) IP_3_ activates IP_3_R on the SR, releasing Ca^2+^. (10–11) Ca^2+^ also activates RyR, amplifying cytosolic Ca^2+^ concentration. (12) Ca^2+^ binds PKC, which is then activated by DAG at the membrane. (13) PKC phosphorylates Ca_V_ channels, (14) enhancing Ca^2+^ influx. (15) The rise in [Ca^2+^]c forms the Ca^2+^–CaM complex, which activates MLCK. (16) leading to phosphorylation of myosin light chains and muscle contraction.

Notably, in some types of smooth muscle, such as uterine smooth muscle, agonists such as acetylcholine can activate receptors coupled to G_i/o_ proteins, causing inhibition of the adenylyl cyclase (AC) pathway and activation of the phospholipase C β2 (PLCβ2) pathway. The decrease in cyclic adenosine monophosphate (cAMP) and the activation of PLC β2 culminate in the contraction of these types of smooth muscle (Figure 5).

In electromechanical coupling, membrane depolarization leads to an increase in [Ca^2+^]_(c)_ due to calcium influx from the extracellular space through voltage-dependent calcium channels (Ca_V_), ultimately initiating the contraction process (Aguilar and Mitchell, 2010; Hill-Eubanks et al., 2011) (Figure 2).

In pharmacomechanical coupling, also referred to as mixed coupling—since it may or may not depend on membrane depolarization—agonists bind to their respective G protein-coupled receptors (GPCRs), activating the inositol signaling cascade via G_q/11_ and/or G_i/o_ proteins. This cascade mediates the production of inositol 1,4,5-trisphosphate (IP_3_), which stimulates calcium (Ca^2+^) release from the sarcoplasmic reticulum, and diacylglycerol (DAG), which activates protein kinase C (PKC). PKC, in turn, promotes an increase in cytosolic calcium concentration ([Ca^2+^]_(c)_), either directly by activating voltage-dependent calcium channels (Ca_V_) or indirectly by inhibiting potassium (K^+^) channels on the plasma membrane (Fukata et al., 2001). The rise in [Ca^2+^]_(c)_ facilitates calcium binding to the protein calmodulin (CaM), forming the active complex [4Ca^2+^–CaM]. This complex activates myosin light chain kinase (MLCK), which phosphorylates the myosin light chain (MLC), promoting its interaction with actin filaments and thereby initiating the contraction process in smooth muscle cells (Webb, 2003) (Figures 4, 5).

**FIGURE 6:**
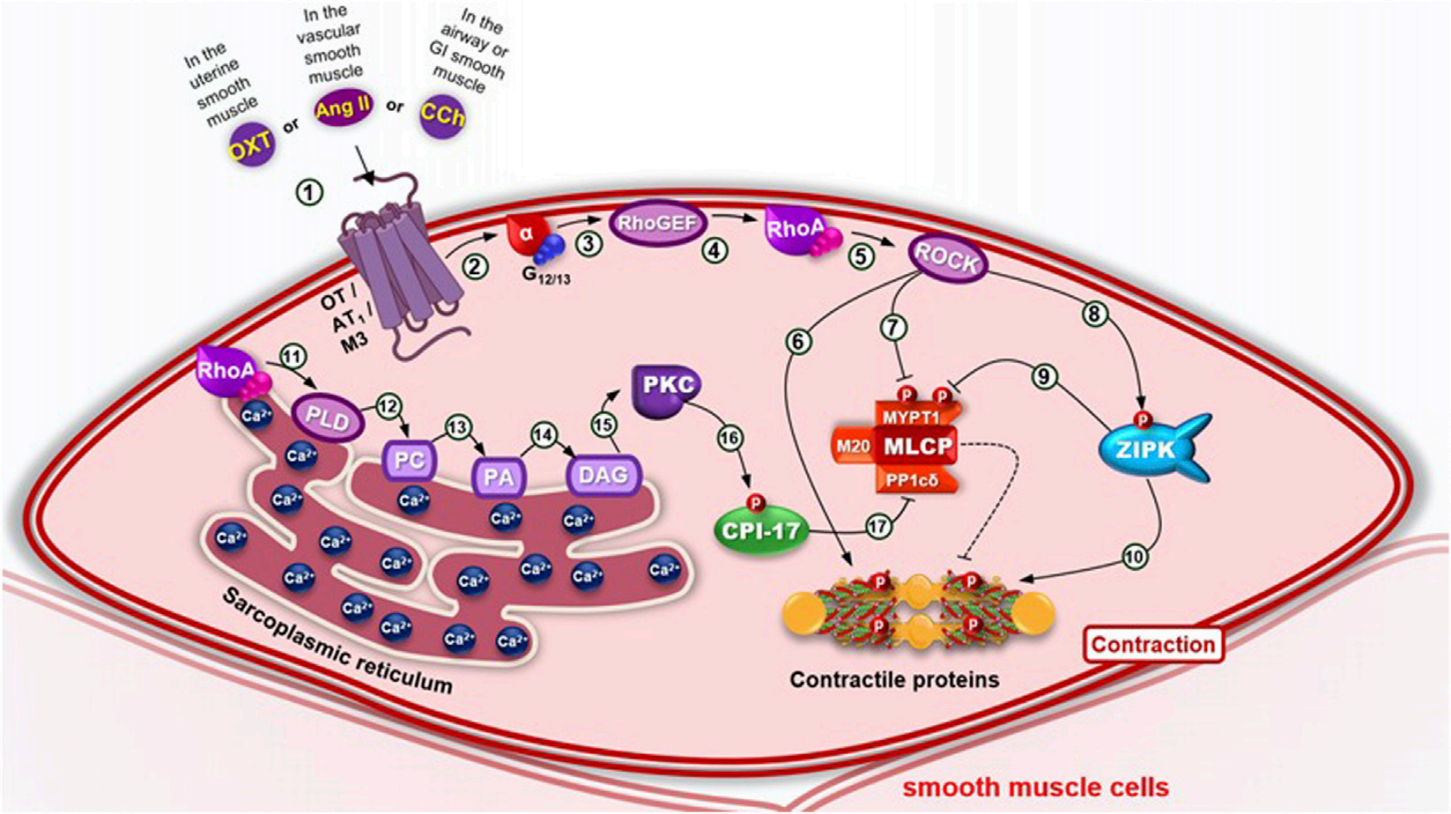
Calcium sensitization pathway in smooth muscle cells. (1) Several agonists—including angiotensin II, norepinephrine and endothelin (in vascular smooth muscle), acetylcholine (in airway or GI smooth muscles) or oxytocin (in uterine smooth muscle) — bind to their respective receptors, triggering a conformational change that recruits G_12/13_ proteins and promotes the exchange of GDP for GTP. (2) This causes steric hindrance and dissociation of the α-GTP subunit from the βγ dimer. (3) The α-GTP subunit activates Rho guanine nucleotide exchange factor (RhoGEF). (4) which exchanges GDP for GTP to activate RhoA, a monomeric G protein. (5) RhoA phosphorylates and activates Rho-associated kinase (ROCK). (6) ROCK phosphorylates the myosin light chain, enhancing actin–myosin interaction; (7) it also phosphorylates and inhibits the catalytic subunit MYPT1 of myosin light chain phosphatase (MLCP), preventing dephosphorylation of contractile filaments. (8) Additionally, ROCK phosphorylates and activates zipper-interacting protein kinase (ZIPK), (9–10) which further inhibits MLCP and promotes myosin light chain phosphorylation. (11) RhoA also activates phospholipase D (PLD), (12) which hydrolyzes phosphatidylcholine (PC) to (13) generate phosphatidic acid (PA), (14) which is converted to diacylglycerol (DAG) by cytosolic phosphohydrolases. (15) DAG activates calcium-independent PKC isoforms, (16) which phosphorylate and activate CPI-17, an inhibitory protein that suppresses the PP1cδ regulatory subunit of MLCP. Inhibition of MLCP through this calcium sensitization pathway enhances smooth muscle contraction.

An alternative pathway contributing to smooth muscle contraction has also been reported, known as the calcium sensitization pathway (Figure 6). Calcium sensitization is a mechanism that is largely independent of intracellular calcium levels and enables modulation of smooth muscle contraction by altering the sensitivity of myosin light chain (MLC) to calcium. This process allows the muscle to sustain contraction even after the initial calcium transient has subsided. Two major mechanisms are involved in calcium sensitization: the diacylglycerol–phospholipase C–protein kinase C (DAG–PLC–PKC) pathway and the RhoA signaling pathway (Lincoln, 2007; Steinberg, 2008).

The calcium sensitization pathway involves the modulation of myosin light chain phosphatase (MLCP) activity by the monomeric G protein RhoA and its associated kinase (ROCK) (Hori and Karaki, 1998). Contractile agonists such as angiotensin II, in vascular smooth muscle, and carbachol (CCh), in airway smooth muscle, activate G_12/13_ proteins generating maintenance of contraction by direct or indirect activation of RhoA-specific guanine nucleotide exchange factors (RhoGEFs), which in turn activate RhoA (Feng et al., 1999; Somlyo and Somlyo, 2003). GTP-bound RhoA activates ROK, which phosphorylates and thereby inhibits MLCP, enhancing MLC phosphorylation mediated by myosin light chain kinase (MLCK), and ultimately promoting smooth muscle contraction (Kimura et al., 1996). Smooth muscle relaxation occurs through a decrease in [Ca^2+^]^c^ (Somlyo and Somlyo, 2003), either via an electromechanical mechanism—characterized by membrane re-polarization or hyperpolarization—or through a pharmacomechanical mechanism, which involves membrane receptor activation and inhibition of the intracellular signaling pathways that drive contraction (Woodrum and Brophy, 2001) (Figure 6).

**FIGURE 7:**
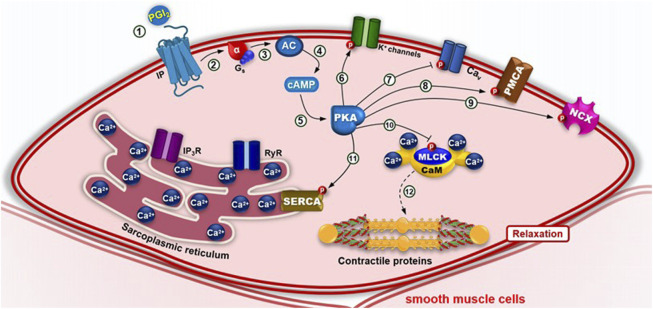
Pharmacomechanical coupling of smooth muscle relaxation via the Gs–adenylyl cyclase (AC) pathway. (1) Agonists ― such as prostacyclin (PGI2) binding to IP receptors (on vascular, uterine, airway or GI smooth muscle) or norepinephrine (NE) binding to β2 receptors (on airway, uterine, GI or vascular smooth muscles) ― bind to their respective receptors, inducing a conformational change that activates the G_s_ protein and promotes GDP–GTP exchange, dissociating the α-GTP subunit from the βγ dimer. (2) The α-GTP subunit activates adenylyl cyclase (AC), (3–4) which converts ATP to cyclic AMP (cAMP). (5) cAMP activates protein kinase A (PKA), which phosphorylates several target proteins: (6) Voltage-dependent potassium (Kv) channels, increasing K^+^ efflux and membrane hyperpolarization; (7) Voltage-dependent calcium channels (Ca_V_), reducing Ca^2+^ influx; (8) Plasma membrane Ca^2+^-ATPase (PMCA; 9) Na^+^/Ca^2+^ exchanger (NCX), lowering cytosolic [Ca^2+^]; (10) MLCK, reducing its activity; and (11) Sarcoplasmic/endoplasmic reticulum Ca^2+^-ATPase (SERCA), enhancing calcium reuptake and (12) promoting smooth muscle relaxation.

**FIGURE 8:**
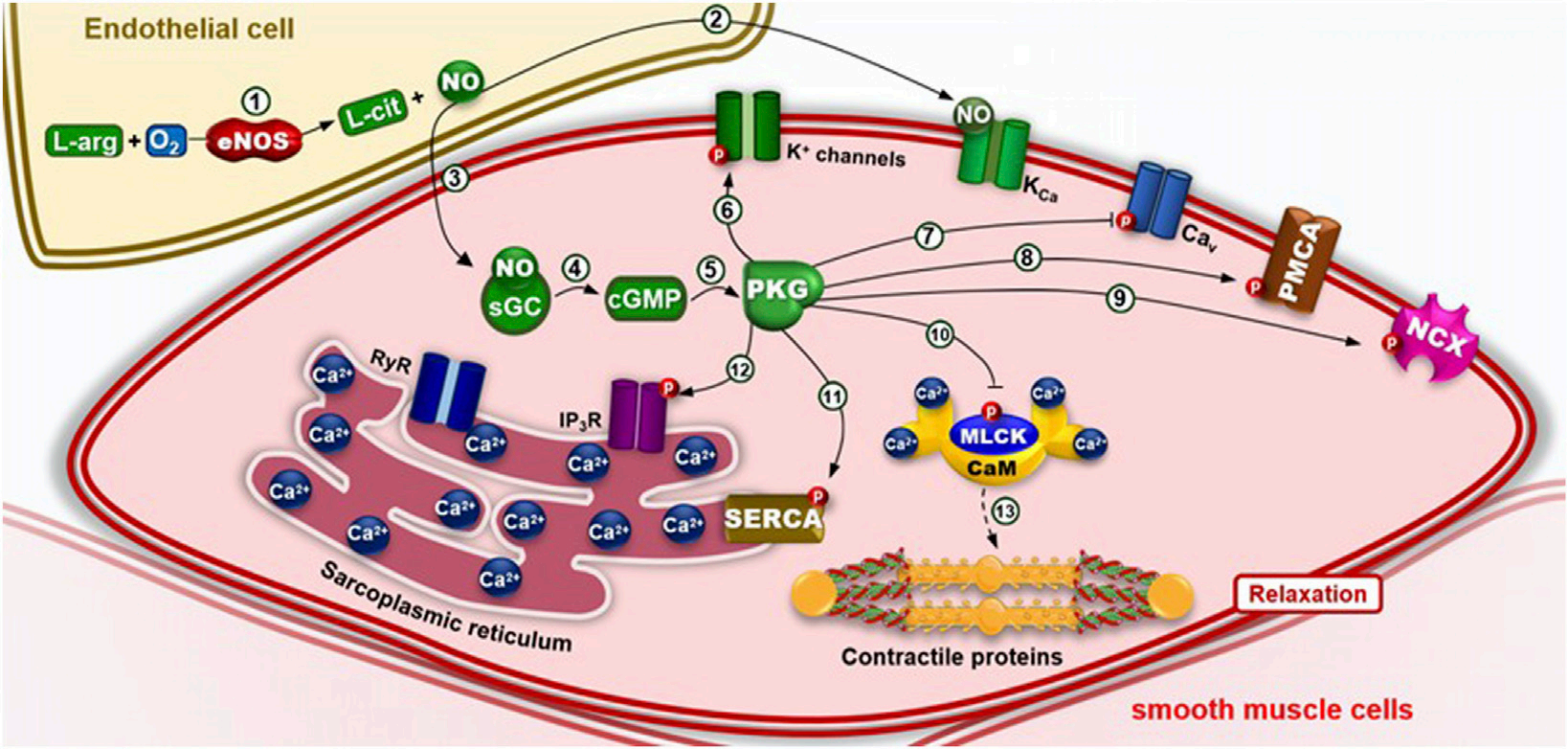
Pharmacomechanical coupling of smooth muscle relaxation via the nitric oxide (NO) pathway. (1) Endothelial nitric oxide synthase (eNOS) catalyzes the oxidation of L-arginine (L- Arg), forming L-citrulline (L-Cit) and NO, in endothelial cells in response to a stimulus by ACh, for example. (2) NO can directly activate calcium-activated potassium (KCa) channels, promoting hyperpolarization and smooth muscle relaxation without requiring downstream signaling. (3) Alternatively, NO diffuses into smooth muscle cells and activates soluble guanylyl cyclase (sGC), (4) which catalyzes the conversion of GTP to cyclic GMP (cGMP). (5) cGMP activates protein kinase G (PKG), which phosphorylates: (6) Kv channels, enhancing K^+^ efflux and membrane hyperpolarization; (7) CaV channels, reducing calcium influx; (8) PMCA and (9) NCX, reducing [Ca^2+^]_(c)_; (10) MLCK, inhibiting contraction; (11) SERCA, enhancing calcium storage; and (12) IP_3_ receptors (IP_3_R), (13) thereby inhibiting calcium release from the sarcoplasmic reticulum and (14) promoting smooth muscle relaxation.

In addition to the general mechanisms involved in smooth muscle contraction, there are also specific pathways responsible for smooth muscle cell relaxation (Figures 7, 8). The main signaling cascades that mediate smooth muscle relaxation involve the second messenger’s cyclic adenosine monophosphate (cAMP) and cyclic guanosine monophos-phate (cGMP). cAMP is generated by adenylyl cyclase (AC) downstream of β-adrenergic or IP receptors coupled to G_s_ proteins, which are activated by norepinephrine or prostacyclin (PGI_2_), respectively. It is noteworthy that while the cAMP pathway typically promotes contraction in cardiac muscle, in smooth muscle, cAMP signaling induces relaxation (Figure 7).

The cGMP pathway can be activated by nitric oxide (NO) or natriuretic peptides (NPs). In blood vessels and other smooth muscle tissues, NO produced by endothelial nitric oxide synthase (eNOS) diffuses across the smooth muscle cell membrane and acti-vates soluble guanylyl cyclase (sGC), which in turn increases intracellular cGMP levels (Figure 8).

## 4 Smooth muscle-related disorders and therapeutic challenges

### 4.1 Asthma

Asthma is a chronic, heterogeneous pulmonary disease marked by airway inflammation, remodeling, hyperresponsiveness, and variable airflow limitation, leading to symptoms such as coughing, wheezing, dyspnea, and chest tightness (Pelaia et al., 2021; Kardas et al., 2020). Affecting over 339 million people worldwide, it imposes a considerable social and economic burden (Zhang et al., 2025; GBD, 2021, 2025).

Airway smooth muscle (ASM) plays a central role in asthma pathophysiology. Once considered a passive contractile element, ASM is now recognized as a dynamic tissue with immunomodulatory, secretory, and remodeling capacities (Camoretti-Mercado et al., 2021; Van der Velden et al., 2013). In asthmatic individuals, ASM exhibits increased proliferation, hypertrophy, contractility, and cytokine secretion compared with non-asthmatic tissue (Ma et al., 2002; Chambers et al., 2003). ASM also releases inflammatory mediators—cytokines, chemokines, and extracellular matrix proteins—that amplify airway inflammation and remodeling (Lambrecht and Hammad, 2015a; Parikh et al., 2019).

From a mechanistic perspective, smooth muscle is the primary cell type responsible for generating tone and airway contraction in the lungs (Prakash, 2013). Upon stimulation by bronchoconstrictor agonists, there is an increase in intracellular calcium concentration ([Ca^2+^]ᵢ), triggering Ca^2+^–calmodulin binding, MLCK activation, and actin–myosin interactions that ultimately result in contraction (Zhu, 2011). Dysregulation of these calcium signaling pathways contributes to hypercontractility in asthma (Prakash et al., 2009; Bergantin, 2020; Sivaraman and Onyenwoke, 2021).

In summary, ASM is a key driver of asthma pathogenesis through its roles in bronchomotor regulation, inflammation, and remodeling. Understanding its functional plasticity is essential for developing targeted therapies.

### 4.2 Gastrointestinal tract disorders

The gastrointestinal (GI) tract is responsible for managing fluids and processing large quantities of solids and semi-solids that pass through the intestinal lumen. It also plays a central role in secretion, digestion, nutrient absorption, and waste elimination (Le et al., 2021; Ghishan and Kiela, 2012). In this context, GI smooth muscle (GISM) is primarily responsible for generating peristalsis, which facilitates efficient digestion, absorption, and excretion. These functions are regulated by the intrinsic electrical and mechanical properties of smooth muscle, through tonic contractions that maintain organ dimensions against luminal content (e.g., food bolus) and/or through the development of contractile force and muscle shortening (Bitar, 2003).

Contractility in gastrointestinal smooth muscle is a highly integrated process, wherein smooth muscle cells represent the final effectors of modulatory inputs from neurons, interstitial cells of Cajal (ICC), hormones, and paracrine substances (Sanders, 2008). These contractions generate the propulsive force needed to move digesta along the GI tract and ensure proper mixing with digestive enzymes, continuously exposing nutrients to the absorptive mucosal surface (Grundy et al., 1985). Therefore, alterations in GISM contractility are directly implicated in the pathophysiology of major gastrointestinal diseases and disorders. The fundamental basis of GI motility lies in the intrinsic ability of GISM to generate cyclic changes in resting membrane potential, which give rise to spontaneous and rhythmic contractions (Grundy et al., 1985). These contractions are tightly regulated by calcium (Ca^2+^) influx, which plays a key role in initiating GISM contraction.

In intestinal regions characterized by phasic motor patterns (small and large intestines), ICC-driven slow waves bring the membrane potential to the threshold of CaV1.2 channels, triggering action potentials (Morgan et al., 1981; Cannell and Lederer, 1987). In contrast, in areas such as the proximal stomach, the resting potential is near CaV1.2 activation, so depolarizing stimuli easily enhance tone (Sanders, 2008; Morgan et al., 1981). GI motility, especially in the ileum, has been studied with isometric transducers in response to electrical field stimulation (EFS), highlighting its key role in nutrient absorption (Nishiyama et al., 2017; Azuma et al., 2021).

### 4.3 Infertility and erectile dysfunction

Smooth muscle plays a critical role in the pathophysiology of infertility and erectile dysfunction (ED), as it is involved in essential physiological processes required for reproductive and sexual function. Consequently, impaired smooth muscle function may directly disrupt these processes, leading to difficulties in achieving conception or maintaining an erection (Hiremath et al., 2020; Souza et al., 2022). Male infertility is often characterized by a reduced sperm count. Studies have shown that this condition is not only associated with impaired sperm production but also with a decreased number of spermatozoa successfully transported through the male reproductive tract (Hiroshige et al., 2024).

The male reproductive tract is lined with smooth muscle that contracts rhythmically to propel sperm. This intrinsic activity ensures sperm movement, and although the autonomic nervous system does not directly trigger these contractile events, it plays a fundamental role in coordinating and modulating such contractions and other critical components of the sexual response (Klinge et al., 1994; Courtois et al., 2013).

A key contributing factor to infertility is erectile dysfunction (ED), defined as the consistent inability to achieve and/or maintain a penile erection sufficient for satisfactory sexual intercourse, affecting approximately 52% of men (Zhuang et al., 2024; Tchang et al., 2021).

Penile erection is a complex physiological process involving coordinated interactions among the nervous, vascular, and endocrine systems. Cavernosal smooth muscle, located in the erectile tissue of the penis, along with smooth muscle in the walls of small arteries and arterioles, plays a central role in erection. Sexual stimulation—triggered by auditory, visual, olfactory, or cognitive stimuli—is processed by the cerebral cortex and relayed through the parasympathetic pathways of the sacral plexus (Dean and Lue, 2005).

This neural activity stimulates the release of nitric oxide (NO) by non-adrenergic, non-cholinergic (NANC) nerve fibers or acetylcholine (ACh) by parasympathetic cholinergic neurons. NO enhances cyclic guanosine monophosphate (cGMP) synthesis and reduces intracellular calcium (Ca^2+^) levels, promoting relaxation of smooth muscle cells. Concurrently, compression of the submucosal venous plexus decreases venous outflow, allowing blood to be retained in the sinusoids of the corpora cavernosa, which progressively increases rigidity and results in full penile erection (Zhuang et al., 2024).

Therefore, smooth muscle is a critical component of the mechanisms underlying both infertility and erectile function. Research into agents capable of modulating smooth muscle activity represents a promising approach for the treatment of related disorders.

### 4.4 Uterine dysfunctions

The uterus is structurally composed of three layers: the endometrium (luminal surface), the myometrium (smooth muscle layer), and the perimetrium (serosal surface) (Hong, 2023; Zhai et al., 2020; Ameer and Munakomi, 2022). The myometrium, the predominant layer, contains longitudinal and circular smooth muscle fibers and is responsible for uterine contractility, essential for menstruation, implantation, and parturition (Wray and Prendergast, 2019).

Among the most prevalent uterine smooth muscle disorders are leiomyoma (uterine fibroids) and adenomyosis, both associated with infertility and abnormal uterine bleeding (Bazot et al., 2001; Stewart et al., 2016; Ahmad et al., 2023). Leiomyomas are benign monoclonal tumors of smooth muscle origin with high prevalence, affecting over 70% of women (Wray and Prendergast, 2019).

Primary dysmenorrhea (PD), the most common gynecological disorder among women of reproductive age, is characterized by cyclic pelvic pain in the absence of underlying pelvic pathology (Itani et al., 2022; Smith et al., 2024). Its pathogenesis is closely linked to elevated levels of prostaglandins—particularly PGF2α—triggered by lysosomal destabilization and increased phospholipase A2 (PLA_2_) activity following the premenstrual drop in progester-one (Dikensoy et al., 2008; Guimarães and Póvoa, 2020). These prostanoids enhance myometrial contractility and vasoconstriction, resulting in ischemia and pain sensitization (Xie et al., 2020; Dawood, 2006).

Oxidative stress also contributes to PD, and conventional treatments (NSAIDs, hormonal contraceptives) may be ineffective or cause adverse effects (Oladosu et al., 2018; Wang et al., 2024; Zhang et al., 2021), highlighting the need for safer alternatives.

### 4.5 Pulmonary hypertension

Pulmonary hypertension (PH) is a complex vascular disorder characterized by remodeling and narrowing of pulmonary arteries, leading to elevated pulmonary arterial pressure, right ventricular failure, and ultimately death (Gao and Raj). Diagnosis is defined by a mean pulmonary artery pressure ≥25 mmHg, though newer guidelines suggest >20 mmHg when combined with other hemodynamic abnormalities (Simonneau et al., 2019; Huetsch et al., 2019).

Smooth muscle cells play a central role in PH pathogenesis through increased proliferation, migration, and enhanced contractile activity, which contribute to vascular stiffening and obstruction. Additionally, the aberrant muscularization of distal arterioles and excessive production of reactive oxygen species (ROS) exacerbate disease progression by promoting inflammation, oxidative stress, and cellular proliferation (Freund-Michel et al., 2013; Fulton et al., 2017; Nozik-Grayck and Stenmark, 2007). Current therapies target vasomotor tone but fail to prevent proliferation and remodeling, underscoring the need for novel interventions.

## 5 Therapeutic potential of *Arthrospira platensis* in smooth muscle disorders

### 5.1 Airways: anti-inflammatory and bronchodilatory effects

Allergic respiratory diseases such as asthma and rhinitis involve chronic airway inflammation, oxidative stress, and bronchial hyperreactivity, often leading to structural remodeling and smooth muscle dysfunction (Lambrecht and Hammad, 2015b; Figueiredo et al., 2023).

In experimental models, *Arthrospira platensis* supplementation has consistently demonstrated antioxidant, anti-inflammatory, and bronchodilatory effects. In asthmatic rats, oral AP (500 mg/kg) reduced airway inflammation, lowering IL-4, IL-5, IL-13, IgE, and tissue damage induced by ovalbumin and cigarette smoke (Riaz et al., 2022). AP also attenuated bronchial smooth muscle hypercontractility. Brito et al. (2022) showed that AP (150–500 mg/kg) decreased tracheal contractile responses to carbachol and increased NO bioavailability, reinforcing its bronchodilatory potential. A key mediator is phycocyanobilin (PhyCB), a biliverdin-derived chromophore abundant in AP, which inhibits NADPH oxidase and decreases oxidative stress, mechanisms that contribute to downregulation of the RhoA/ROCK pathway involved in smooth muscle contraction (McCarty et al., 2021).

In humans, clinical evidence is still limited. A trial in patients with allergic rhinitis found that *Spirulina* supplementation reduced IL-4 levels (Mao et al., 2005).

Collectively, these data suggest that AP exerts protective effects on airway smooth muscle by reducing hyperresponsiveness and inflammation through antioxidant and NO-mediated pathways. However, these results are restricted to rodent models, and the only available human data derive from an early allergic rhinitis trial with poorly characterized interventions. No randomized controlled trials (RCTs) exist for asthma, and future studies should prioritize standardized preparations, validated biomarkers of airway function, and direct comparisons with bronchodilators or corticosteroids.

### 5.2 Gastrointestinal tract: gastroprotection and motility modulation

Gastrointestinal disorders, including gastritis, ulcers, and reflux, are influenced by multiple factors such as *Helicobacter pylori* infection, inflammation, and oxidative stress (Wroblewski et al., 2010; Xiao et al., 2021). The gastric smooth muscle contributes to motility and mucosal protection, and its dysfunction is often exacerbated by mucosal injury and reactive oxygen species.

Preclinical evidence indicates that AP exhibits gastroprotective properties mediated by its antioxidant and anti-inflammatory metabolites. In rodent models of gastric ulcer induced by indomethacin or diclofenac, AP supplementation reduced mucosal injury, decreased lipid peroxidation (↓ MDA), and increased antioxidant enzymes (↑ SOD, GSH) (Aleid et al., 2021; Nipa et al., 2020). Similarly, in aspirin-induced gastric injury, AP reduced TNF-α and COX-2 expression while enhancing antioxidant defense (Mahmoud and Abd El-ghffar, 2019). Beyond gastroprotection, a polysaccharide fraction from AP selectively inhibited gastric adenocarcinoma proliferation without affecting normal gastric cells (Uppin et al., 2022).

Taken together, these results support the use of AP as a functional candidate for protecting gastric mucosa and modulating smooth muscle dysfunction. Nonetheless, existing studies are highly heterogeneous in design, extract preparation, and dosing, and none have used pharmacological comparators such as omeprazole or misoprostol. Clinical studies remain absent, and pharmacokinetic assessment of active metabolites is required before translation.

### 5.3 Intestine: anti-inflammatory and antioxidant actions

The smooth muscle of the small intestine is essential for peristalsis, nutrient absorption, and intestinal barrier function. Alterations in contractility and redox balance are implicated in inflammatory bowel diseases and obesity-related gastrointestinal dysfunction (Sanders, 2008).

In chemically induced colitis models, AP supplementation reduced disease severity, lowered proinflammatory cytokines (TNF-α, IL-1β, IL-6), and decreased oxidative markers (Fado et al., 2020). In obese rats, AP (25 mg/kg) prevented intestinal contractile dysfunction by modulating voltage-dependent calcium channels and downregulating muscarinic M3 receptors (Diniz et al., 2021). Other studies confirmed that AP modulates the RhoA/ROCK pathway, nitric oxide signaling, prostanoids, and superoxide dismutase activity in intestinal smooth muscle (Diniz et al., 2024). In Wistar rats, AP (150–500 mg/kg) reduced ileal contractile responses to carbachol and KCl while enhancing antioxidant activity (Araújo et al., 2016).

Clinical evidence is scarce but promising. In a randomized trial with patients with irritable bowel syndrome (IBS), AP supplementation improved antioxidant capacity, reduced malondialdehyde (MDA), and improved intestinal permeability and symptom severity (Jafari Nasab et al., 2025).

Thus, evidence from animal models and one clinical trial suggests that AP exerts intestinal protective effects by reducing inflammation, preserving barrier integrity, and preventing contractile dysfunction. However, standardized preparations, dose–response studies, and comparisons with reference drugs are lacking.

### 5.4 Corpus cavernosum: erectile function and reproductive health

*Arthrospira platensis* has shown promising effects on disorders involving the corpus cavernosum, particularly infertility and erectile dysfunction (ED), often-linked to metabolic conditions such as obesity. Experimental studies in animal models have demonstrated that AP supplementation not only improves erectile function but also promotes overall male reproductive health.

Preclinical studies show that AP supplementation improves erectile function under metabolic stress conditions. In obese rats with diet-induced ED, oral AP (25 mg/kg, 8 weeks) increased erection frequency, reduced latency to first erection, and improved relaxation responses in penile tissue (Diniz et al., 2020; Souza et al., 2022). These effects were absent in healthy animals, suggesting context-specific benefits. Mechanistically, AP enhances NO bioavailability, suppresses contractile prostanoids, and reduces oxidative stress, improving smooth muscle relaxation and vascular function (Souza et al., 2022).

Beyond erectile function, AP demonstrates protective effects on the male reproductive system against environmental toxins. Studies report improvements in sperm count, motility, steroid hormone levels, and reduced testicular oxidative damage following exposure to agents such as arsenic, lead, and cyclophosphamide (Afkhami-Ardakani et al., 2021; Bashandy et al., 2016; El-Hakim et al., 2018). Furthermore, the seaweed improved steroid hormone production and spermatogenesis in rats exposed to the toxin, further reinforcing its role in male reproductive health (El-Desoky et al., 2013; Farag et al., 2016; Ibrahim et al., 2021).

These findings indicate that AP may act as an adjunct therapy for ED and infertility, especially in obesity and toxic exposures. Nevertheless, no clinical trials are available, extract standardization is lacking, and positive pharmacological comparators (e.g., PDE5 inhibitors) have not been used in animal models.

### 5.5 Uterus: dysmenorrhea and uterine tone modulation

Uterine smooth muscle, primarily located in the myometrium, plays a central role in menstruation, fertility, and parturition. Dysfunctions in its contractility are associated with disorders such as primary dysmenorrhea (PD), adenomyosis, and uterine fibroids (Wray et al., 2019; Stewart et al., 2016).

*Arthrospira platensis* has demonstrated therapeutic potential in modulating uterine smooth muscle activity, largely due to its antioxidant and anti-inflammatory properties. In experimental models, AP supplementation (50–100 mg/kg) prevented increases in contractile reactivity to KCl and oxytocin in rats undergoing resistance training, while preserving relaxation responses to nifedipine and isoprenaline. These effects were associated with enhanced nitric oxide signaling, inhibition of prostanoid pathways, and reduction in reactive oxygen species (Ferreira et al., 2021; Barros, 2021).

Khatun et al. (2018), K et al. (2019) demonstrated that AP (200 mg/kg) protected uterine and ovarian tissues from arsenic-induced oxidative damage by boosting endogenous antioxidant enzymes (SOD, catalase, peroxidase). In a PD rat model, AP reduced oxytocin-induced writhing and prevented hypercontractility by modulating oxidative stress and prostaglandins (Lacerda-Júnior, 2022; Soares, 2025; Melchiades Júnior, 2024). Additionally, AP extract and its major pigment, C-phycocyanin, exhibited direct spasmolytic effects on pre-contracted guinea pig uterus, likely via L-type calcium channel inhibition (Marangoni et al., 2017; Bannu et al., 2019).

Altogether, these results support AP as a potential therapy for uterine contractility disorders, particularly PD. However, all evidence derives from animal studies with small sample sizes, no comparators with standard uterotonics or spasmolytics, and no clinical validation.

### 5.6 Vasculature: vascular reactivity and blood pressure control

Vascular smooth muscle (VSM) is essential for regulating vascular tone, blood pressure, and vessel remodeling. Dysfunction in VSM contractility and proliferation is implicated in hypertension, atherosclerosis, and endothelial dysfunction. *Arthrospira platensis* has shown promising vasoprotective effects in both functional and molecular studies.

Brito et al. (2018) demonstrated that AP supplementation (150–500 mg/kg) improved aortic reactivity in Wistar rats, reducing contractile responses to phenylephrine and enhancing acetylcholine-induced relaxation. These effects were associated with increased nitric oxide (NO) bioavailability and reduced lipid peroxidation. The NO synthase inhibitor L-NAME reversed these effects, confirming the involvement of the NO pathway.

In spontaneously hypertensive rats (SHR), aqueous extracts of Spirulina platensis promoted vasorelaxation via enhanced NO production, without affecting superoxide levels. This was accompanied by upregulation of endothelial proteins such as AKT and heme oxygenase-1 (HO-1), which are essential for NO synthesis and oxidative protection (Villalpando et al., 2020; Castro-García et al., 2018).

In preeclampsia models, phycobilins (100 mg/kg/day) lowered systolic blood pressure and increased eNOS expression (Castro-García et al., 2018). C-phycocyanin inhibited vascular smooth muscle cell proliferation *in vitro* by upregulating cell-cycle inhibitors p21 and p27 (Xianming, 2013). Translational potential is supported by a triple-blind, placebo-controlled clinical trial, where daily consumption of Spirulina-enriched dressing improved vascular function in humans (Ghaem Far et al., 2025).

Overall, these findings provide robust preclinical evidence and some preliminary clinical support for AP’s vasoprotective effects. However, extract standardization is poor, trials remain small, and pharmacological comparators with standard antihypertensives are missing.

## 6 Therapeutic and preventive role of *Arthrospira platensis* in obesity

Obesity is a chronic, multifactorial inflammatory condition characterized by excessive fat accumulation and associated with comorbidities such as type 2 diabetes, hypertension, cardiovascular diseases, and certain cancers, significantly reducing quality and life expectancy (Khanna et al., 2022). Its prevalence has grown substantially worldwide, with projections from the World Health Organization estimating 2.3 billion overweight adults and 700 million obese individuals by 2025 (PAHO, 2023). This trend is largely driven by nutritional transition, including increased intake of ultra-processed foods and reduced physical activity (Mialon et al., 2021).

Due to the limitations of conventional treatments, there is increasing interest in alternative approaches involving functional natural products. *Arthrospira platensis*, a nutrient-rich cyanobacterium, has shown promise in reducing body fat, improving lipid profiles, and preserving nutritional status in obesity (Silva et al., 2023).

Studies in obese Wistar rats fed a hypercaloric diet demonstrated that *S. platensis* supplementation (50 mg/kg for 8 weeks) restored erectile function, reduced oxidative stress, increased nitric oxide (NO) availability, and preserved penile vascular endothelium (Souza et al., 2022). At higher doses (500 mg/kg for 8 weeks), it reduced malondialdehyde (MDA) levels and hepatic lipid deposition, suggesting antioxidant and hepatoprotective effects (Arrari et al., 2023).

Yu et al. (2020) showed that S. platensis (3% dietary inclusion for 14 weeks) significantly reduced body weight, visceral fat, serum lipopolysaccharides (LPS), and pro-inflammatory cytokines (IL-6, TNF-α, IL-1β) in rats. It also modulated gut microbiota composition, lowering the Firmicutes/Bacteroidetes ratio and improving intestinal barrier function by increasing expression of tight junction proteins such as ZO-1, occludin, and claudin-1.

Additional research confirmed that *S. platensis* (50 mg/kg for 8 weeks) restored erectile function by increasing NO bioavailability, reducing ROS production, enhancing total antioxidant capacity, and normalizing acetylcholine-induced relaxation, highlighting its endothelium-modulating potential (Diniz et al., 2020; Diniz et al., 2021). Moreover, supplementation at 25 mg/kg prevented obesity development and preserved intestinal reactivity in rats, reducing oxidative stress and IL-1β expression (Diniz et al., 2023).

Taken together, recent evidence supports the pharmacological potential of *A. platensis* in managing obesity and related disorders. Its effects are attributed to antioxidant, anti-inflammatory, immunomodulatory actions, and its rich nutritional composition, reinforcing its value as a functional supplement.

## 7 Limitations and future perspectives

Although substantial preclinical evidence supports the therapeutic effects of *Arthrospira platensis (AP)* on smooth muscle dysfunction across multiple organ systems, several important research gaps remain to be addressed. First, most findings to date are derived from animal models or *in vitro* studies, with limited clinical validation. Well-designed, placebo-controlled human trials are necessary to confirm the efficacy, safety, and appropriate dosing regimens of AP for conditions such as asthma, dysmenorrhea, erectile dysfunction, and gastrointestinal or vascular disorders. Second, the precise molecular targets and signaling pathways modulated by AP remain partially understood. While existing studies highlight the involvement of nitric oxide bioavailability, calcium channel regulation, antioxidant systems, and the RhoA/ROCK and prostaglandin pathways, further mechanistic investigations are required to delineate tissue-specific effects and interactions with conventional pharmacological agents. Third, the bioavailability and pharmacokinetics of AP’s active metabolite—such as phycocyanin, phycocyanobilin, and polysaccharides—need deeper exploration to determine their absorption, metabolism, and systemic impact in humans. Additionally, standardization of AP extracts with defined chemical profiles would ensure consistency and reproducibility across studies and clinical applications. Lastly, the therapeutic potential of AP in complex conditions such as metabolic syndrome, obesity-related reproductive dysfunction, and inflammatory bowel diseases suggests its utility as a multifunctional intervention. Future research should investigate its use in combination with other nutraceuticals or pharmaceuticals, as well as its long-term safety profile. Altogether, advancing from preclinical promise to clinical implementation will require integrative research efforts bridging molecular pharmacology, clinical nutrition, and translational medicine.

To provide a comprehensive overview of the available evidence, we summarized the pharmacological and clinical studies evaluating *A. platensis* in smooth muscle-related disorders. Table 1 outlines the experimental models, doses, extract types, controls applied, main findings, and limitations reported in each study. This structured presentation allows for a clearer assessment of the methodological quality and translational relevance of the available data.

**TABLE 1:** Critical appraisal of preclinical and clinical studies evaluating *Arthrospira platensis* in smooth muscle-related disorders.

Author / Year	Experimental model (*in vitro/in vivo*/clinical)	Dose/Concentration	Type of extract/fraction	Controls used	Main findings	Limitations
Brito et al., 2022	Wistar rats, isolated trachea (*in vivo*)	150–500 mg/kg (oral)	Lyophilized powder	KCl, carbachol, verapamil	Reduced tracheal contractility; ↑ NO production	No clinical validation; translational dose unclear
Khatun et al., 2018	Female rats, arsenic induced uterine toxicity (*in vivo*)	200 mg/kg (oral)	Aqueous extract	Control + arsenic group	Protection of uterine and ovarian tissues; ↑antioxidants	Toxicity model only; no natural dysfunction
Diniz et al., 2024	Obese rats, isolated ileum (*in vivo/in vitro*)	25 mg/kg (oral) 8 weeks	Standardized aqueous extract	KCl, carbachol, L- NAME	Prevented ileal dysfunction via RhoA/ROCK and NO	Preclinical only; short duration
Yu et al., 2020	Obese rats, dietary intervention (in vivo)	3% diet for 14 weeks	Whole biomass	Normal and obese controls	Reduced obesity, inflammation, improved microbiota	Did not directly assess smooth muscle contractility
Enkhmaa et al., 2006	Clinical trial, metabolic syndrome patients	2 g/day	Commercial *Spirulina* powder	Placebo	Improved lipid profile and blood pressure	Small sample; smooth muscle not directly evaluated
Brito et al., 2019	Wistar rats, isolated aorta (in vivo/in vitro)	150–500 mg/kg	Aqueous extract	Phenylephrine, acetylcholine, L-NAME	↑ Endothelium dependent relaxation via NO	No chemical standardization
Brito et al., 2022	Rats, tracheal smooth muscle (in vivo)	150–500 mg/kg	Powder/extract	Carbachol, NO inhibitors	Reduced airway hyperreactivity, bronchodilation	Rodent-only; lack of standardized
Clinical trial, rhinitis (human) (137)	Clinical trial, allergic rhinitis patients	2 g/day	Commercial *Spirulina* powder	Placebo	Reduced IL-4 in allergic rhinitis	Small trial; no extract standardization
Gastroprotection models (140-142)	Rodent ulcer models (indomethacin, diclofenac, aspirin)	50–500 mg/kg	Aqueous extracts	NSAIDs injury models	Reduced mucosal damage, lipid peroxidation, ↑ antioxidants	Heterogeneous extracts; no pharmacological comparators
Anti-neoplastic effect (143)	Gastric cancer cell model	Polysaccharide fraction	Polysaccharide	Normal gastric cells	Inhibited gastric adenocarcinoma proliferation	In vitro only; no animal/human validation
Clinical trial, IBS (145)	Clinical trial, IBS patients	Daily supplementation (dose NR)	Not reported	Placebo group	Improved antioxidant status, reduced IBS symptoms	Small sample; limited characterization of product
Obese rats, Ileum contractility (146-147)	Wistar rats, ileal reactivity (in vivo)	25–500 mg/kg	Aqueous extract	KCl, carbachol	Reduced ileal contractility, ↑ antioxidants	No reference drugs; lack of chemical standardization
Corpus cavernosum studies (50-53, 148-153)	Rats, penile tissue and sperm parameters	25–200 mg/kg, 8 weeks	Whole biomass/extract	Obese vs control rats	Improved erectile function, sperm quality, ↓ oxidative damage	No PDE5 inhibitor comparators; no clinical studies
Uterine models (156-164)	Rats, uterine hypercontractility and toxin exposure	50–200 mg/kg	Extract and phycocyanin	Oxytocin, KCl, nifedipine	Prevented uterine hyperreactivity, ↑NO, ↓ ROS	Limited to animal studies; no clinical validation
Vascular studies SHR/aorta (165-170)	SHR rats, vascular reactivity studies	150–500 mg/kg	Aqueous extracts	Phenylephrine, acetylcholine, L-NAME	Improved vascular relaxation, ↑NO bioavailability	Small trials; extract composition not standardized
Preeclampsia model (168)	Rat preeclampsia model	100 mg/kg/day	Phycobilins	Normotensive vs preeclampsia	Reduced BP, ↑eNOS expression	Animal-only; needs human validation
C-phycocyanin proliferation study (169)	In vitro VSMC proliferation assay	Purified -C-phycocyanin	C-phycocyanin	Untreated VSMCs	Inhibited VSMC proliferation via p21/p27	In vitro only; lacks clinical translation
Clinical trial, vascular function (170)	Triple-blind human vascular study	2 g/day	*Spirulina*-enriched dressing	Placebo dressing	Improved vascular function in humans	Small clinical trial; exploratory
Obesity rodent models (51, 147, 175-176)	Obese rats, multiple tissues (ileum, penis, liver, microbiota)	25–500 mg/kg; 3% diet;	Whole biomass/extract	Obese vs lean rats	↓ Oxidative stress, ↓ inflammation, improved organ function	Lack of dose standardization; no long-term safety data

NR, not reported; SHR, spontaneously hypertensive rat; IBS, irritable bowel syndrome; VSMC, Vascular smooth muscle cell. Limitations reflect methodological issues such as lack of standardization, absence of comparators, or insufficient clinical validation.

As shown in Table 1, most of the available data derive from preclinical *in vivo* models, with marked heterogeneity in extract preparation, doses tested, and outcome measures. Only one small clinical trial was identified, which did not directly assess smooth muscle function. These limitations highlight the need for well-designed studies with standardized preparations, robust pharmacological evaluation, and controlled clinical trials to clarify the therapeutic potential of *Arthrospira platensis* in smooth muscle-related pathologies.

## 8 Conclusion

Smooth muscle plays a central role in regulating physiological processes across multiple organ systems, and its dysfunction contributes significantly to the pathogenesis of respiratory, gastrointestinal, reproductive, and cardiovascular disorders. This review highlights *A. platensis* as a promising multifunctional agent capable of modulating smooth muscle function through antioxidant, anti-inflammatory, and immunomodulatory mechanisms. Evidence from preclinical studies consistently demonstrates that A. platensis supplementation improves smooth muscle contractility and mitigates pathological remodeling in various tissues, including the airways, gut, vasculature, uterus, and corpus cavernosum. These beneficial effects appear to be mediated by key molecular pathways involving nitric oxide bioavailability, calcium signaling, prostaglandin modulation, and oxidative stress regulation. Nevertheless, despite these encouraging experimental results, the clinical translation of *A. platensis* remains limited due to the lack of robust human trials. Further investigations are required to clarify its pharmacokinetics, establish optimal dosing regimens, and evaluate its long-term safety profile in humans. In conclusion, A. platensis represents a valuable candidate for the development of integrative therapeutic strategies targeting smooth muscle–related diseases. Its application as a natural functional supplement may offer a safe and effective complement to conventional pharmacotherapy, particularly in chronic inflammatory and metabolic conditions.

## Glossary

ACh: Acetylcholine
AC: Adenylyl Cyclase
ALT: Alanine Aminotransferase
AMPK: AMP-Activated Protein Kinase
AP: Arthrospira platensis
ASM: Airway Smooth Muscle
AST: Aspartate Aminotransferase
ATP: Adenosine Triphosphate
cAMP: Cyclic Adenosine Monophosphate
Ca2+: Calcium Ion
CaM: Calmodulin
CaV: Voltage-Dependent Calcium Channel
COX-2: Cyclooxygenase-2
cGMP: Cyclic Guanosine Monophosphate
DAG: Diacylglycerol
eNOS: Endothelial Nitric Oxide Synthase
ED: Erectile Dysfunction
EFS: Electrical Field Stimulation
GISM: Gastrointestinal Smooth Muscle
GPCR: G Protein-Coupled Receptor
GPx: Glutathione Peroxidase
GR: Glutathione Reductase
GSH: Reduced Glutathione
IL: Interleukin
IP3: Inositol 1,4,5-Trisphosphate
IP3R: IP3 Receptor
iNOS: Inducible Nitric Oxide Synthase
K+: Potassium Ion
Kv: Voltage-Gated Potassium Channel
L-NAME: NG-Nitro-L-arginine methyl ester (NOS inhibitor)
LPS: Lipopolysaccharide
MAPK: Mitogen-Activated Protein Kinase
MDA: Malondialdehyde
MLCK: Myosin Light Chain Kinase
MLCP: Myosin Light Chain Phosphatase
MLC: Myosin Light Chain
MMP: Matrix Metalloproteinase
NE: Norepinephrine
NF-κB: Nuclear Factor Kappa B NO: Nitric Oxide
NOS: Nitric Oxide Synthase
Nrf2: Nuclear Factor Erythroid 2–Related Factor 2
PD: Primary Dysmenorrhea
PG: Prostaglandin
PGF2α: Prostaglandin F2 Alpha
PhyCB: Phycocyanobilin
PLA2: Phospholipase A2
PLC: Phospholipase C
PLD: Phospholipase D
PKA: Protein Kinase A
PKC: Protein Kinase C
PKG: Protein Kinase G
PMCA: Plasma Membrane Ca2+-ATPase
PUFA: Polyunsaturated Fatty Acid
RhoA: Ras Homolog Family Member A
ROCK: Rho-Associated Protein Kinase
ROS: Reactive Oxygen Species
RyR: Ryanodine Receptor
SHR: Spontaneously Hypertensive Rat
SIP Syncytium: Smooth Muscle–Interstitial Cell–PDGFRα+ Syncytium
SMC: Smooth Muscle Cell
SOD: Superoxide Dismutase
SR: Sarcoplasmic Reticulum
TNF-α: Tumor Necrosis Factor Alpha
ZIPK: Zipper-Interacting Protein Kinase
ZO-1: Zonula Occludens-1
